# Supplementary material for: Inferring the stabilization effects of SARS-CoV-2 variants on the binding with ACE2 receptor
Source: Commun Biol. 2022 Jan 6;5:20221. doi: 10.1038/s42003-021-02946-w (PMC8738749; doi:10.1038/s42003-021-02946-w)
Supplement: Supplementary file 2 — Supplementary Information [file 42003_2021_2946_MOESM2_ESM.pdf]

# Supplementary Information: Inferring the stabilization effects of SARS-CoV-2 variants on the binding with ACE2 receptor

Mattia Miotto,[1] Lorenzo Di Rienzo,[1] Giorgio Gosti,[1] Leonardo Bo',[1] Giacomo Parisi,[1] Roberta Piacentini,[2,1] Alberto Boffi,[3,1] Giancarlo Ruocco,[1,4] and Edoardo Milanetti[4,1]

[1] *Center for Life Nano & Neuroscience, Istituto Italiano di Tecnologia, Viale Regina Elena 291, 00161, Rome, Italy*
[2] *Department of Biochemical Sciences "Alessandro Rossi Fanelli", Sapienza University of Rome, P.Le A. Moro 5, 00185, Rome, Italy*
[3] *Department of Biochemical Sciences "Alessandro Rossi Fanelli", Sapienza University of Rome, P.Le A. Moro 5, 00185, Rome, Italy*
[4] *Department of Physics, Sapienza University, Piazzale Aldo Moro 5, 00185, Rome, Italy*

## I. ADDITIONAL TABLES AND FIGURES

| residue 1 | residue 2 | K417N − N501F | K417T − N501F |
|---|---|---|---|
| 417 | 455 | 0.20 | 0.22 |
| 417 | 456 | 0.18 | 0.22 |
| 417 | 493 | 0.16 | 0.09 |
| 484 | 501 | 0.16 | 0.13 |
| 493 | 501 | 0.21 | 0.09 |
| 494 | 501 | 0.24 | 0.16 |
| 495 | 501 | 0.21 | 0.19 |
| 496 | 501 | 0.28 | 0.23 |

Supplementary Table I: Correlations between the motion of the couples of residue of the binding region of the spike protein involving either residue 417 and/or 501 in the modified South African and modified Amazonian variant complexes.

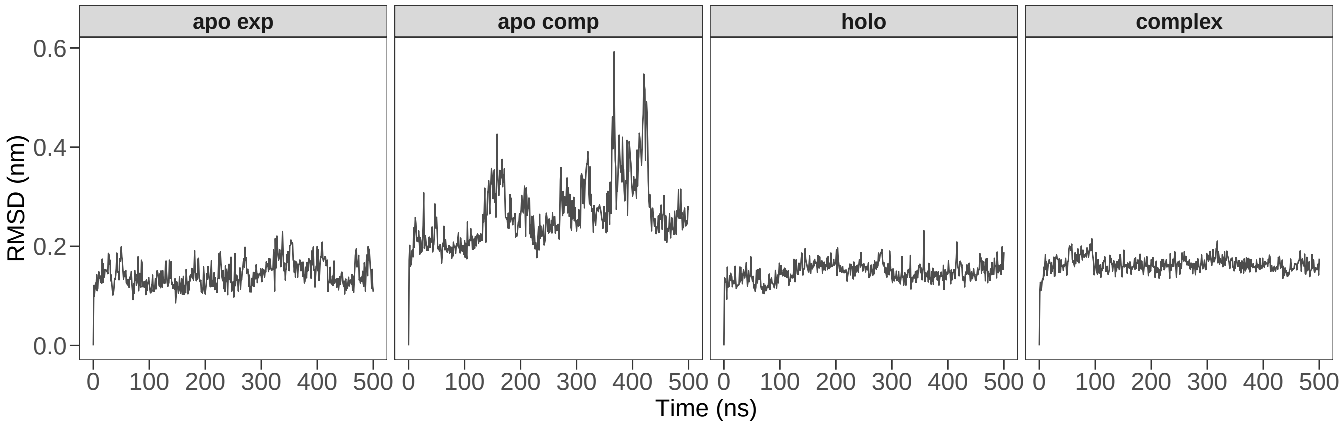


Supplementary Figure 1: Root mean square deviation (RMSD) of the experimental apo conformation (pdb id: 7LYN), modeled apo conformation (pdb id: 7KJ5), modeled holo conformation (pdb id: 6M0J) and modeled conformation in complex (pdb id: 6M0J) of the SARS-CoV-2 spike of the South African variant (Mutations: K147N_E484K_N501Y).

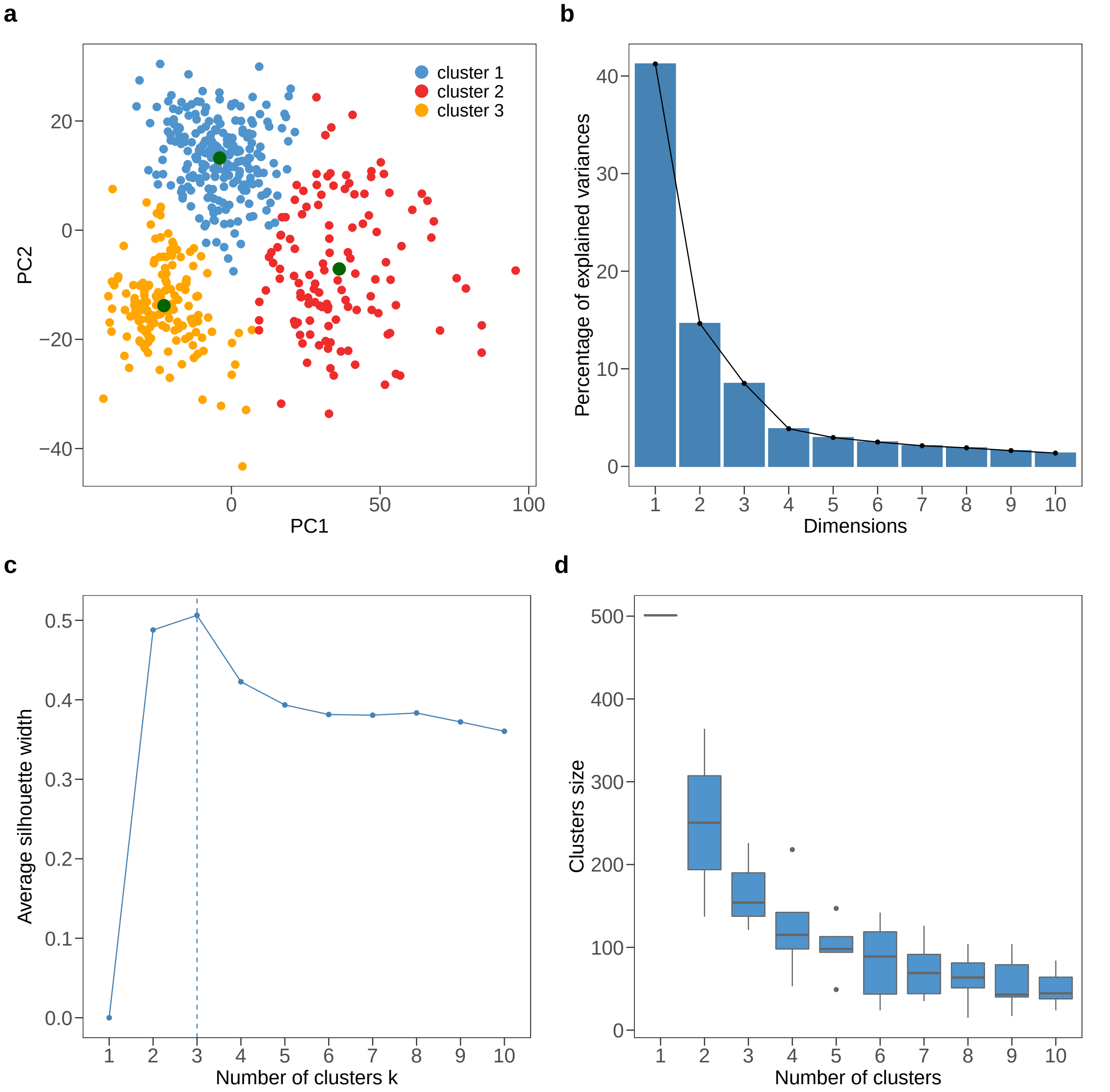


Supplementary Figure 2: **a)** Projection of a representative set of configurations of the computationally-mutated SARS-CoV-2 spike in the apo state on the plane identified by the first two principal components of the covariance matrix. Clustering analysis identified three clusters, whose centroids are represented with dark green dots. **b)** Percentages of explained variances for the first ten principal components. The first two components cover over 65% of the total variance. **c)** Average silhouette as a function of the number of clusters. The maximum is reached for $n = 3$ clusters which correspond to the division in panel a). **d)** Boxplot of the number of points belonging to each cluster as a function of the number of clusters.

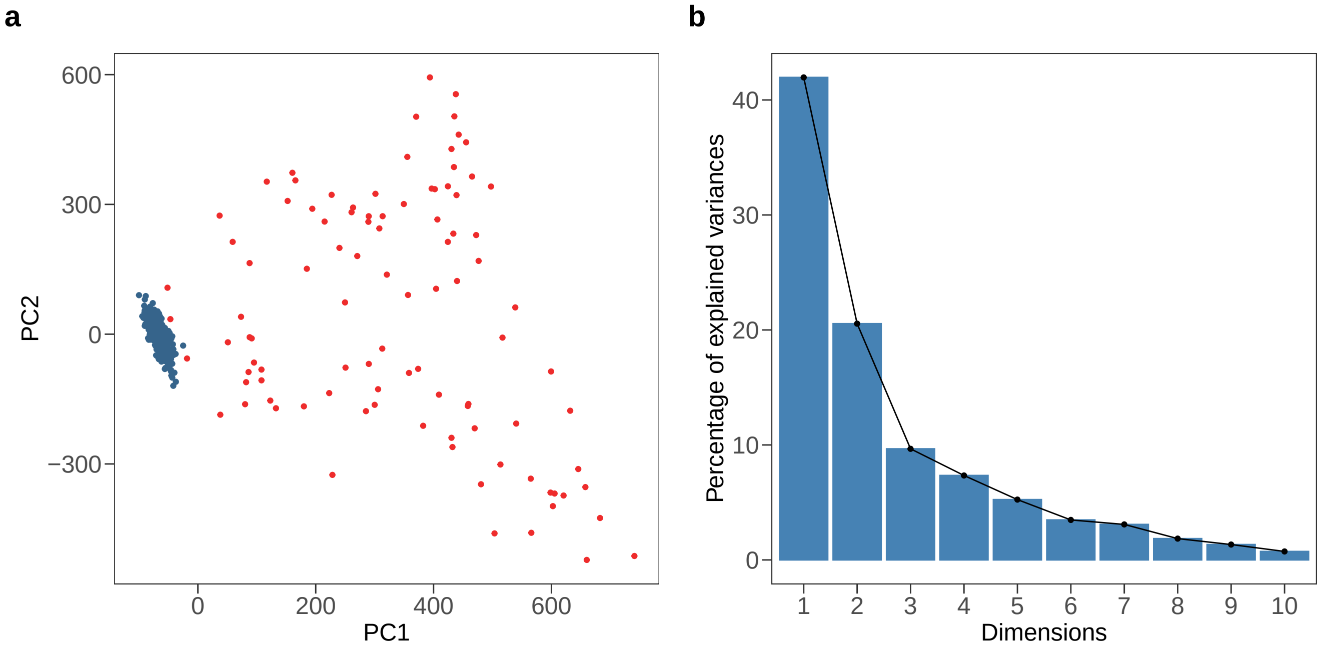


Supplementary Figure 3: **a)** Projection of a representative set of configurations of the computationally-mutated South African variant of SARS-CoV-2 spike on the plane identified by the first two principal components of the covariance matrix (blue dots). Red dots correspond to the top 100 docking poses obtained starting from the first centroid of the spike in apo form (see Supplementary Figure 2a) and the human ACE2 receptor. **b)** Percentages of explained variances for the first ten principal components. The first two components cover over 60% of the total variance.

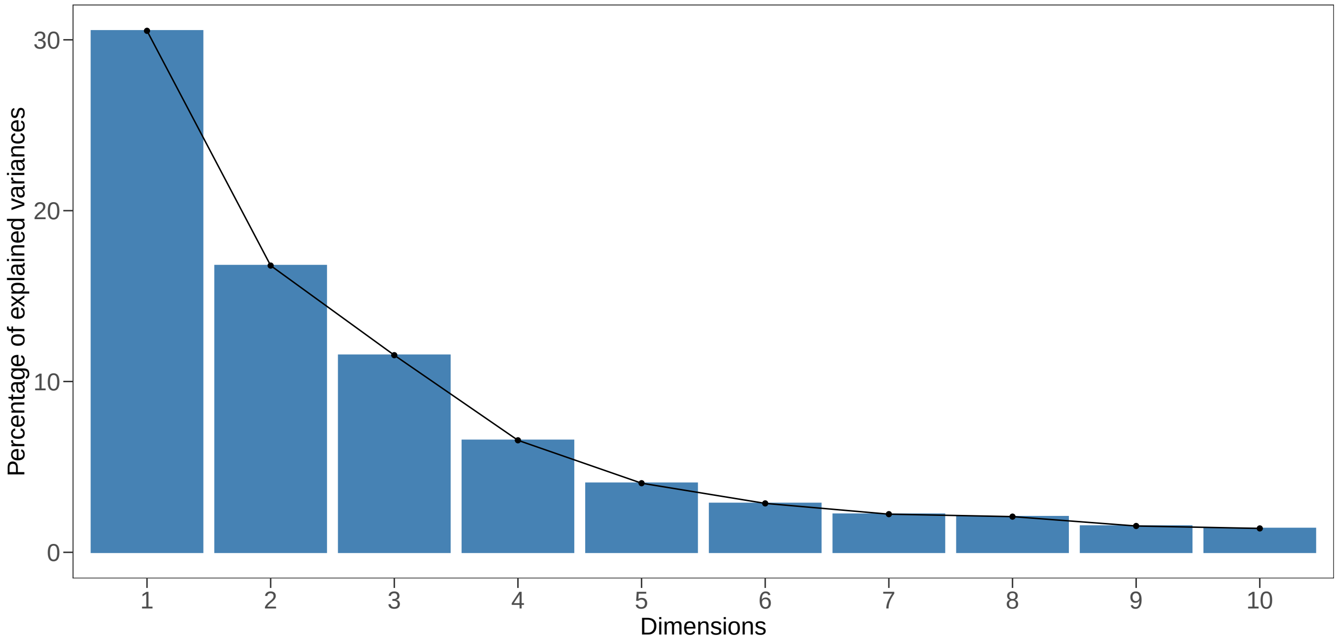


Supplementary Figure 4: Percentages of the explained variances for the first ten principal components of the PCA performed on the English variant (mutation: N501Y variant) simulation in Figure 2a of the Main Text. The first two components cover over 45% of the total variance.

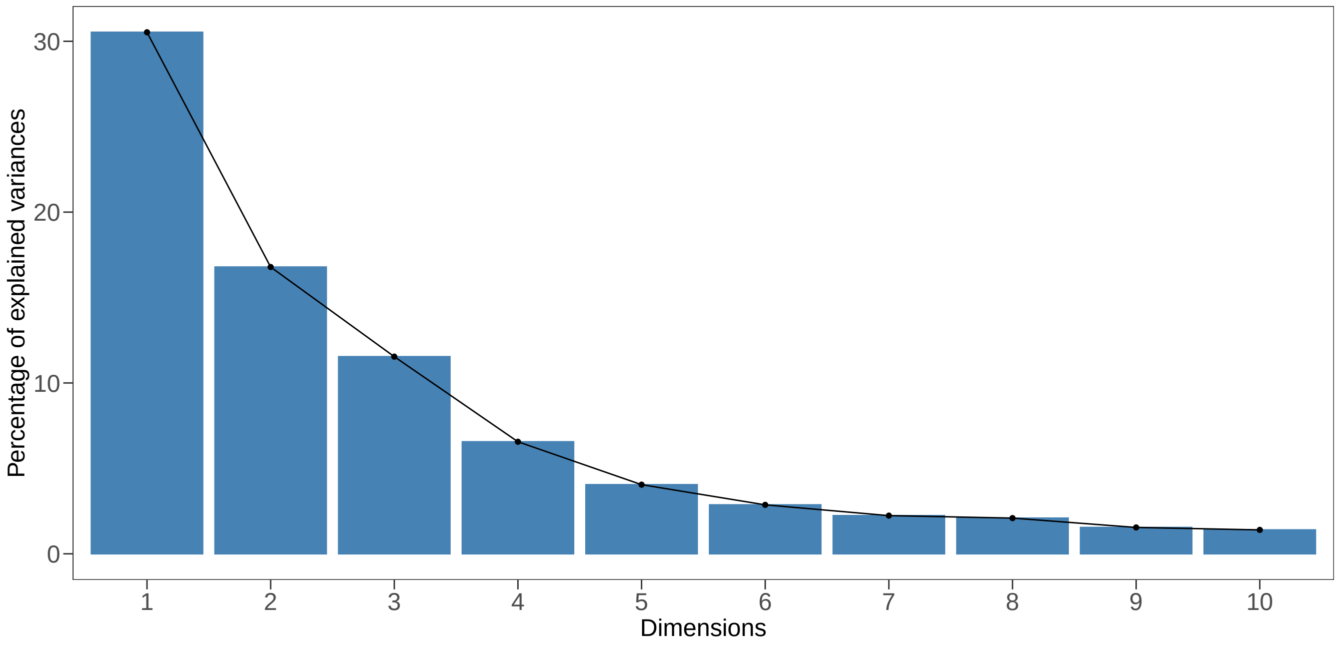


Supplementary Figure 5: Percentages of the explained variances for the first ten principal components of the PCA performed on the Amazonian variant (mutation: K417T_E484K_N501Y) simulation in Figure 2b of the Main Text. The first two components cover over 45% of the total variance.
